# Surgical Treatment Combined With Concentrated Growth Factor Injection for a Case of Pseudopelade of Brocq

**DOI:** 10.1111/jocd.70101

**Published:** 2025-03-04

**Authors:** Miaoqi Qiu, Yujie Miao, Zhongfa Lv, Jing Jing

**Affiliations:** ^1^ Department of Dermatology, Second Affiliated Hospital Zhejiang University School of Medicine Hangzhou China

**Keywords:** concentrated growth factor, primary cicatricial alopecia, pseudopelade of Brocq, surgical treatment

Dear Editor,

A 29‐year‐old Chinese woman presented with multiple alopecia patches persisting for over 20 years following trauma. She had no prior treatment or history of systemic disease or skin disease. Examination revealed three round alopecic patches on the vertex and parietal scalp. Dermoscopy showed ivory‐white, shiny skin with no follicular openings, scaling, or inflammation (Figure 1A). A hair pull test was negative. Based on clinical and dermoscopic findings, a diagnosis of Pseudopelade of Brocq (PPB) was made. Given the stability and localized nature of the lesions, scalp reduction surgery was performed. Histopathological analysis (Figure 2) confirmed the absence of hair follicles and sebaceous glands, replaced by fibrous tissue extending into the epidermis and subcutaneous fat, consistent with PPB. Two months postoperatively, the patient developed erythema, itching, and scar hyperplasia at the surgical site (Figure 1B); then, monthly injection of concentrated growth factor (CGF) was initiated. After six injections, the erythema and itching were relieved, the scar healed well, and the surrounding area of the surgical site showed significant improvement, with a hair density of 98.763 hairs/cm^2^ observed under dermoscopy (Figure 1C). Additionally, no other medications were used. PPB is a scarring alopecia which is clinically similar to alopecia areata (AA) but without the potential for hair regrowth. Current treatment mirrors that of lichen planopilaris, with oral JAK inhibitors or hydroxychloroquine recommended during active disease. For stable cases, surgical interventions such as scalp reduction or hair transplantation are considered. However, surgical complications like hyperplastic scarring and ischemic necrosis must be carefully managed to avoid alopecia recurrence [1]. CGF is rich in growth factors, including transforming growth factor‐beta 1 (TGF‐β1), vascular endothelial growth factor (VEGF), and CD34‐positive cell populations, which promote hair follicle stem cell proliferation and differentiation while regulating endothelial cell barrier function to suppress inflammation [2]. Additionally, CGF exhibits antifibrotic potential [3], suggesting that early CGF treatment may inhibit scarring and prevent atrophy. CGF has shown efficacy in promoting hair regrowth and restoring hair density in non‐scarring alopecia like androgenetic alopecia and alopecia areata [4]. In a patient with scarring alopecia secondary to discoid lupus erythematosus, CGF injection also demonstrated satisfactory clinical results [5]. Our case highlights that surgical excision combined with CGF injection for stable, localized PPB is effective and innovative, and also more economical and safer than hair transplant. Based on existing evidence, it is recommended to initiate CGF therapy 10 days post‐surgery, followed by injections every 2–4 weeks [4].

**FIGURE 1:**
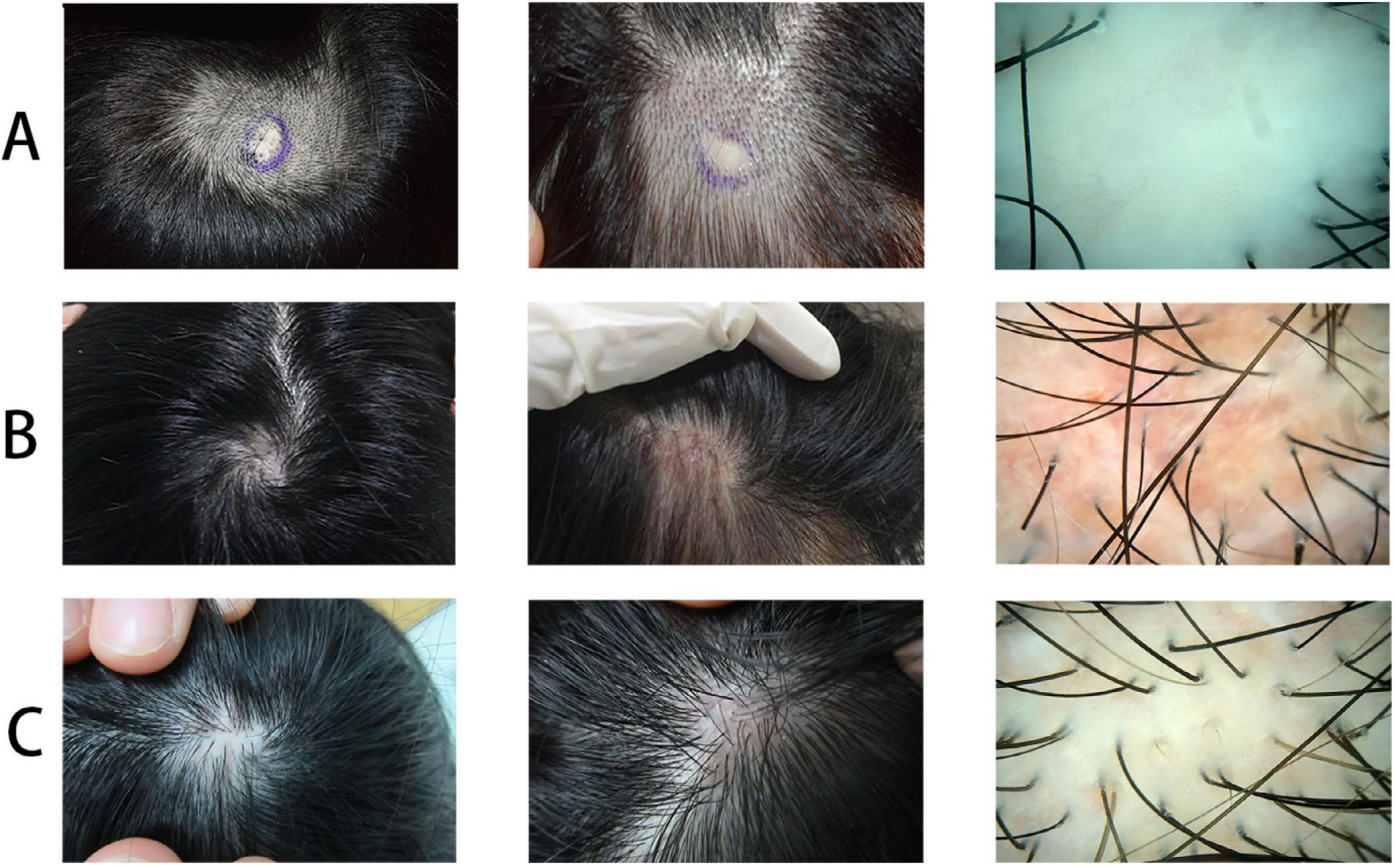
Sequential lesion and dermoscopy progression during treatment. (A), Preoperative skin lesions and dermoscopy. (B), Postoperative erythema and scarring. (C), Scalp condition after six CGF injections.

**FIGURE 2:**
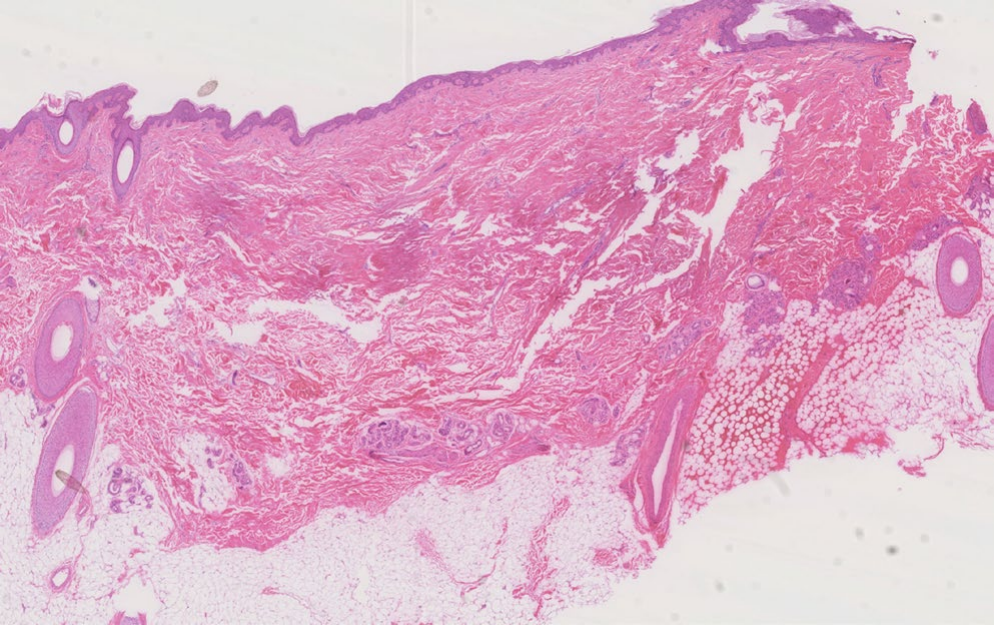
The pathology of Pseudopelade of Brocq.

## Author Contributions

**Miaoqi Qiu:** conceptualization, methodology, investigation, formal analysis, writing – original draft; **Yujie Miao:** data curation, writing original – draft; **Zhongfa Lv:** visualization, investigation; **Jing Jing:** resources, conceptualization, supervision, writing – review and editing.

## Consent

Consent for the publication of recognizable patient photographs or other identifiable material was obtained by the authors and included at the time of article submission to the journal, stating that all patients gave consent with the understanding that this information may be publicly available.

## Conflicts of Interest

The authors declare no conflicts of interest.

## Data Availability

The authors have nothing to report.
